# Labourers' Wages and Labourers' Homes

**Published:** 1888-05-19

**Authors:** Earl Grey


### Labourers' Wages and Labourers' Homes.

By the Right Honourable Earl Grey, K.G., G.C.M.G.

*(Continued from p. 88.)*

Most, if not all, the schemes brought forward for diminishing the distress of the unemployed poor of London propose for that purpose to increase the demand for labour by undertaking works of public utility. Those schemes differ from each other as to what works might be undertaken with most advantage, and as to the manner in which the money required for their execution should be provided, but they agree in looking for the relief of those of the London poor for whom sufficient employment cannot now be found mainly to the creation of an additional demand for their labour. To all schemes of this kind it has been objected that the evil to be dealt with cannot be thus corrected, and would, on the contrary, be increased in the end, if not immediately, by the adoption of any of them. The employment that would be afforded by the proposed works would attract candidates for it from a distance at least as numerous as those of the present London population, to whom it would afford a resource, and as the employment thus artificially created could not be permanent, when it ceased there would probably be a greater number of labourers out of work to be provided for than before. What has happened in Paris affords an instructive warning of the danger of attempting to relieve a working population from the distress arising from a want of employment by creating an artificial demand for labour. The magnificent improvements made in the French capital during the reign of the last Emperor were undertaken, as is well known, mainly for the purpose of affording employment to the many artizans and labourers who suffered from difficulty in finding work. The failure of this policy was anticipated by men of the greatest experience and knowledge of France, and, as it turned out, with but too much reason. Before long it became clear that, while more and more money was spent in trying to relieve the unemployed by creating an artificial demand for labour, the difficulty of the task thus rashly undertaken continued to increase, and each year brought with it a need for still larger sums than before in order to carry on the policy which had been entered upon. The war of the Commune, and the present uneasy condition and dangerous temper of the largest part of the Parisian population are in no small degree to be attributed to this mistaken policy.

The objections urged against the scheme of seeking to relieve the unemployed labourers of London by undertaking public works for the purpose of creating a better demand for their labour have been met by proposals that employment on these works should only be given to men who could show that they had been living in London for not less than one or two years, or by paying less than current wages to the labourers. By either of these means it has been said that the danger of attracting additional labourers to London by undertaking the works suggested would be averted; but if I am not mistaken, the last of these two proposals has been condemned (and not without reason) by the working men who have considered the subject as tending to lower the wages now earned by labourers in the same sort of work as that which would be thus underpaid, and both proposals are open to the obvious objection that wages paid under such conditions would in fact be given as relief instead of as the legitimate earnings of industry. Thus the works would at once assume the character of relief works with all the dangers of abuse to which such works are exposed, and the men employed upon them would feel that they were the recipients of charity, and not maintaining themselves by independent industry. The demoralizing effect of placing them in this position is too well understood to require to be insisted upon. In what I have just said I do not mean to deny that it may be expedient in special cases and in order to meet a temporary necessity to have recourse to relief works; occasions, no doubt, arise when it is almost impossible to avoid doing so; but to trust to such works for the purpose of dealing with a chronic deficiency of employment which regularly recurs at certain seasons of the year is a very different matter, and would, as I believe, be attended with very great evil.

The conclusion I come to is, therefore, that a remedy for the admitted evils and dangers arising from the present condition of a part of the London population, must be looked for in quite a different direction from that in which it has lately been sought. I would venture to suggest that what ought to be aimed at is to check the excessive resort to London of more candidates for employment than are likely to obtain it under conditions consistent with really civilized life, instead of persevering in a vain attempt to relieve the struggling competitors for insufficient opportunities for obtaining work from the difficulties and privations they have now to endure, either by charity or by measures for creating an artificial demand for labour. I call this a vain attempt, for it is proved to be so as well by the acknowledged failure of the large subscriptions obtained by the Lord Mayor's Fund and otherwise for the relief of London to do any permanent good, as by the more disastrous failure of the national workshops in Paris after the revolution of 1848, and of the measures of the late Emperor of France.

On the other hand, if means could be found to check the excessive resort to London of those who are in search of employment, although no great immediate change for the better in the condition of the present working population of the metropolis could be expected to follow, the greatest obstacle to the success of other measures for improving that condition would be got rid of, since nothing has so powerfully contributed to keep down the general scale of living of this population, and to compel them to dispense with the decent comforts of civilised life as the continual influx into London of additional candidates for employment. The too great increase of the population of London, from its attracting so many new inhabitants from a distance, has been a matter of complaint from early times, and in the reigns of the Tudors and the Stuarts some feeble and ineffectual attempts were made to restrain it by authority. These attempts do not seem to have been renewed at a later period, but from time to time the subject has engaged the attention of writers on public affairs; and about seventy years ago Cobbett used to denounce in his *Register* with characteristic energy the growth of what he called the "Wen" of the nation as one of the crying evils of the day. There was great exaggeration in his complaints, for with the development of the nation and the increase of its wealth and population the enlargement of the capital became necessary. Still, it must be admitted that his denunciations of the "Wen" had a foundation in truth, and even in his day there were clear indications that the increase of the population of London in the manner in which it was allowed to go on, without restraint or regulation, was already producing very serious evils, though they were not then felt with anything approaching to their present intensity. In the present state of things no doubt can be entertained that it is desirable, if possible, to check the excessive resort to London from foreign countries, as well as from our own, of those who are in search of employment, of which enough is not to be found there both for the present population and for sc many new comers. The question therefore to be considered is whether any effectual and unobjectionable means can be found of preventing so large a number of seekers for work from flocking to London? This question is not the same with regard to foreigners and to subjects of the Queen. The latter, it must at once be admitted, could not justly be forbidden to seek a home and employment in London when they think fit to do so, nor could they when there be required to submit to any restrictions upon the freedom of their actions beyond those applying to other persons. But without infringing upon the rights of British subjects, it does not seem impossible that something at least might be done to discourage the resort to London of those whose going there is likely to prove injurious to themselves as well as to others.

It has been suggested, and the suggestion seems to be a valuable one, that a good deal might be effected in this direction by a stricter enforcement of existing laws against the building or occupation of houses in a manner objectionable on sanitary grounds, assisted by some additional legislation to make these laws more really effective than they now are. By various Acts passed in the last thirty or forty years, Parliament intended to prevent houses from being used as dwellings when likely to prove injurious to the health of their inmates and of the public in consequence of faults either in their construction or in the manner in which they are occupied. Much good has been done by these Acts, and especially by that for the regulation of common lodging-houses, but their intended object is very far indeed from having been accomplished, and it is notorious that there are a very great number of what are called "tenement houses" inhabited by a large population in reckless disregard of the requirements of health and of decency. The failure of the laws which have been passed to guard against this evil is due in part to their provisions not being sufficiently stringent, but still more to the fact that enough care had not been taken to secure their being enforced. The business of enforcing them is in many cases entrusted to local authorities such as vestries, boards of guardians, and boards of health, and unfortunately it not uncommonly happens that these bodies fall under the influence of persons who as owners or lessees of land or houses are interested in the abuses it is their duty to put down. Even when the local authorities have been anxious to perform their duty they have been much hindered in their endeavours to do so by the want of less costly and more effectual means than now exist of dealing with breaches of the law.

(*To be continued.*)

